# The role of the intrinsic pathway of apoptosis in human ejaculated sperm damage under a state of scrotal heat stress

**DOI:** 10.1007/s10815-023-02992-9

**Published:** 2023-12-08

**Authors:** Marta Budzinska, Marzena Kamieniczna, Lukasz Wojnar, Kamil Gill, Malgorzata Piasecka, Michal Kups, Monika Fraczek

**Affiliations:** 1 Institute of Human Genetics, Polish Academy of Sciences, Strzeszynska 32, 60-479 Poznan, Poland; 2 Clinic of Urology and Oncological Urology, Poznan University of Medical Sciences, Poznan, Poland; 3 Department of Histology and Developmental Biology, Faculty of Health Sciences, Pomeranian Medical University in Szczecin, Szczecin, Poland; 4 Department and Clinic of Urology and Oncological Urology, Regional Specialist Hospital in Szczecin, Szczecin, Poland; 5 The Fertility Partnership Vitrolive in Szczecin, Szczecin, Poland

**Keywords:** Male infertility, Genital heat stress, Oxidative stress, Sperm apoptosis

## Abstract

**Purpose:**

The study aimed to determine the associations among standard sperm characteristics and oxidative/apoptotic markers in ejaculated sperm of men exposed to prolonged scrotal hyperthermia of either environmental or clinical origin.

**Methods:**

The original study design included four research groups: professional drivers (*n* = 54), infertile men with varicocele (*n* = 78), infertile men not exposed to prolonged genital heat stress (*n* = 37), and fertile individuals serving as the control group (*n* = 29). Standard semen analysis was performed according to the 5th WHO laboratory manual. The following oxidative and apoptotic parameters of sperm were investigated: mitochondrial superoxide anion generation (MitoSOX Red dye), phosphatidylserine externalization (Annexin V binding assay), mitochondrial membrane potential (JC-1 dye), DNA fragmentation (TUNEL/PI assay), and membrane fluidity (merocyanine 540 dye).

**Results:**

All the studied groups presented a strong deterioration in routine sperm parameters and a strongly apoptotic phenotype in sperm, characterized by both decreased mitochondrial membrane potential and enhanced DNA fragmentation, regardless of the thermal insult. Significant induction of mitochondrial superoxide anion generation was noted only in the groups exposed to genital heat stress. A positive correlation between the production of superoxide anion in the mitochondrial chain and the level of DNA fragmentation in drivers was also noted.

**Conclusion:**

Long-term exposure to scrotal hyperthermia in real-life situations is sufficient to reduce sperm quality in humans. The thermal stress directly induces the oxidative stress cascade in ejaculated sperm, affecting the plasma membrane fluidity, mitochondrial homeostasis, and sperm DNA integrity.

**Supplementary Information:**

The online version contains supplementary material available at 10.1007/s10815-023-02992-9.

## Introduction

Mammalian testes and epididymides require temperatures between 2 and 7 °C below that of the body’s core temperature to support effective sperm production and maturation. Long-term exposure to scrotal heat stress may disturb or even inhibit the course of spermatogenesis [1, 2]. Animal models designed to investigate the effects of testis heating indicate the vulnerability of the male germ line (in a range of cell types) to thermal insult, especially of round spermatid and pachytene spermatocyte populations [3]. Moreover, experimental approaches have provided evidence to support the presence of oxidative stress in the testis under thermal conditions that may be directly involved in a variety of death processes in germ cells, including apoptosis, necrosis, autophagy, and cell cycle arrest [3–5]. Redox imbalance is also thought to be involved in the pathogenesis of ejaculated sperm damage induced by heat; however, it should be emphasized that the molecular events observed in reproductive organs and semen may vary. Although experimental models have been very useful for studying the pathophysiological mechanisms responsible for germ cell disorders induced by heat stress, we need to be careful when extrapolating such results to spermatogenesis and semen quality in humans.

A majority of retrospective studies have shown the adverse effects of internal factors associated with raised scrotal temperature on standard semen variables. Decreasing sperm number accompanied by loss of sperm progressive motility and morphology were the most frequent sperm alterations revealed in ejaculated sperm attributable to varicocele [6], cryptorchidism [7], infection with febrile episodes [8], and obesity [9]. However, the involvement of thermogenic factors in the aforementioned clinical pathologies has not been well assessed. There is a growing interest in the effect of environmental scrotal heat stress on male fertility. Men subjected to thermal factors related to lifestyle (e.g., sitting or sleeping postures, tight clothing, sauna, hot bath) occupation (e.g., sedentary work, welders, ceramic oven operations) are predisposed to an increased scrotum temperature and weakened standard sperm characteristics [10–12]. Furthermore, a few clinical experimental studies conducted so far in men subjected to transient scrotal warming demonstrated changes in nonconventional semen parameters, especially with respect to oxidative stress and apoptotic markers, such as seminal malondialdehyde concentration [13], seminal soluble Fas [14], caspase-3 activity in sperm [15], and/or sperm DNA fragmentation index [14, 16]. However, none of them have exclusively focused on the signatures of both processes in sperm cells. Moreover, the significance of oxidative stress and apoptosis for male subfertility/infertility resulting from real-life exposure to genital heat stress has not been established.

In light of the above findings, current knowledge on the pathophysiology of human subfertility/infertility caused or complicated by genital heat stress needs to be updated through new clinical retrospective studies including a range of analyzed parameters conducted on groups of men exposed not only to clinical factors but also to environmental thermogenic factors. Over 40 years ago, a higher incidence of subfertility/infertility in heat-exposed occupational drivers and a gradual decline in sperm production with driving experience were revealed [17]. The results of subsequent studies also suggested that a prolonged driving position might be a risk factor for sperm quality, but no molecular research explaining the pathomechanisms has been done [18, 19]. Recently, we undertook an originally designed study that for the first time offered an integrated analysis of network parameters examining the status of semen (both sperm and seminal plasma) in professional drivers, infertile men with varicocele, and infertile men not exposed to prolonged genital heat stress [20, 21]. In the present study, we focused on cellular and molecular sperm characteristics that are hallmarks of oxidative stress and apoptosis, which are probably the key processes associated with heat-induced ejaculated sperm injury. This study provides a clinical verification of the influence of the oxidative stress cascade on sperm characteristics previously demonstrated in animal heat stress models [4].

## Material and methods

### Study groups

The research was conducted in a population of 198 men of reproductive age (median age: 32.50; range (20–41)). Between January 2017 and November 2020, the subjects were recruited from the Andrology Outpatient Clinics in Poznan and Szczecin and via traditional and social media advertisements using the same protocols and following the same exclusion criteria as previously established [20, 21]. Briefly, all the patients and volunteers underwent andrological and ultrasound examinations. A questionnaire about fertility status, genitourinary and systemic diseases, medications, occupation, and lifestyle was completed by all the participants. Individuals with active systemic or genitourinary inflammation, cryptorchidism, hypogonadism, testicular injury or cancer, body mass index > 32 kg/m^2^, a smoking habit, or double local temperature factor were excluded. The participants were divided into four groups: (A) men with proven fertility (at least one offspring over the past 2 years) not exposed to prolonged genital heat stress, serving as the controls, *n* = 29; (B) men who had worked at least 2 years as professional drivers, *n* = 54; (C) infertile men (no pregnancy after at least 1 year of regular unprotected sexual intercourse without any apparent reason in their partner(s)) with clinically diagnosed varicocele (dilatation of the vessels of the pampiniform plexus at least 3 mm in diameter), *n* = 78; and (D) infertile men not exposed to prolonged (clinical or environmental) genital heat stress, *n* = 37.

### Manual semen analysis

Semen samples were collected before any medical intervention. Three to 5 days of sexual abstinence were asked of the participants before ejaculates were collected by masturbation. After liquefaction at room temperature, standard semen analysis was done according to the 5th WHO laboratory manual within 60 min of ejaculation by a trained technician [22]. After initial macroscopic assessment of the samples (color, viscosity, volume, and pH), microscopic assessment was performed under a bright-light microscope equipped with a contrast phase (DM 2000, Leica, Heerbrugg, Switzerland). The sperm concentration was estimated with an improved Neubauer hemocytometer (Paul Marienfeld, Lauda-Königshofen, Germany). Motility characteristics were evaluated using the standard grading system: progressive motility, nonprogressive motility, and immotility. Both eosin staining and hypo-osmotic swelling (HOS) tests were used to assess sperm viability. Sperm morphology was assessed according to Kruger’s strict criteria following Papanicolaou staining of the previously washed sperm smears. To distinguish peroxidase-positive leukocytes from peroxidase-negative round cells (other round cells), the LeucoScreen Kit (FertiPro N.V., Beernem, Belgium) was used.

### Preparation of sperm suspensions

Collected semen samples were centrifuged at 1800 rpm for 7 min at room temperature. The sperm pellets were washed in warm phosphate-buffered saline (PBS), pH 7.4, by centrifugation at 1800 rpm for 7 min. The fresh sperm suspensions were immediately used for fluorescence and flow cytometry studies. To assess sperm DNA fragmentation, an aliquot of sperm suspension was fixed in 1% formaldehyde at 4 °C for 20 min. After washing two times in PBS, the fixed sperm cells were resuspended in ice-cold 75% (v/v) ethanol and stored at − 20 °C for the TUNEL assay for no less than 3 months [21].

### Evaluation of membrane fluidity

The level of phospholipid scrambling in the sperm membrane lipid bilayer was evaluated by merocyanine 540 (M540) staining [23]. Sperm cells were incubated with 4.09 µmol merocyanine 540 (M540) dye (Ex/Em 563/607 nm, emission of red fluorescence) for 15 min in darkness at 37 °C in a CO_2_ atmosphere. Then, flow cytometry analysis was performed (see below). The subpopulation of sperm cells without membrane lipid disorders (M540-negative sperm) was calculated.

### Evaluation of PS translocation

Phosphatidylserine (PS) externalization in sperm membranes was evaluated by using the Annexin V-FITC Apoptosis Detection Kit (Beckman Coulter, Fullerton, CA, USA) according to the manufacturer’s guidelines [23]. Sperm cells washed in PBS supplemented with Ca^2+^ and Mg^2+^ were incubated with Annexin V-FITC and propidium iodide (PI) for 15 min in darkness on ice. Then, flow cytometry analysis was performed (see below). The subpopulation of apoptotic sperm cells (Annexin V-positive and PI-negative sperm) was calculated.

### Evaluation of mitochondrial membrane potential

The mitochondrial membrane potential of sperm was evaluated by using lipophilic cationic 5,5′,6,6′-tetrachloro-1,1′,3,3′-tetraethylbenzimidazol-carbocyanine iodide JC-1 staining [24]. Sperm cells were incubated with 10 µg/ml JC-1 dye (Ex/Em490/529 nm, emission of green fluorescence of JC-1 monomers and Ex/Em 514/590 nm, emission of orange fluorescence of JC-1 aggregates) for 25 min in darkness at 37 °C in a CO_2_ atmosphere. After washing in warm PBS, sperm smears were evaluated under a fluorescence microscope (Olympus BX41, Tokyo, Japan) equipped with a triple emission filter (DAPI/FITC/Texas Red) at × 1000 magnification. The subpopulation of sperm cells with polarized mitochondria (JC-1–positive sperm) was calculated.

### Detection of mitochondrial superoxide anion

Mitochondrial superoxide anion generation in sperm was evaluated by using MitoSOX Red staining [25]. Sperm cells were incubated with 2 µmol MitoSOX Red dye (Ex/Em 561/603, emission of red fluorescence) for 15 min in darkness at 37 °C in a CO_2_ atmosphere. After washing three times in warm PBS, flow cytometry analysis was performed (see below). The subpopulation of sperm cells that emitted red fluorescence (MitoSOX Red-positive sperm) was statistically calculated.

### Evaluation of DNA fragmentation

Nuclear DNA strand breaks in sperm were evaluated by using the FlowTACS Apoptosis Detection Kit (Trevigen, Inc., Minneapolis, MN, USA) by the TUNEL/PI method [26]. The previously fixed (see above) and permeabilized (0.1% Triton X-100 in 0.1% sodium citrate) sperm cells were incubated with the labeling solution containing 1 × binding buffer, biotinylated dNTPs, Mn^2+^, and terminal deoxynucleotidyl transferase (TdT) enzyme (not in negative control) for 45 min at 37 °C. Then, the sperm pellet was incubated with FITC-labeled streptavidin solution for 20 min at room temperature. After the TUNEL labeling reaction, sperm cells were stained with PI to discriminate apoptotic cells from necrotic cells in the flow cytometry analysis. The subpopulation of sperm with DNA fragmentation (TUNEL-positive sperm) was calculated.

### Flow cytometry analysis

Cytofluorometric evaluation of sperm suspensions was performed using a Beckman Coulter flow cytometer (Cell Lab Quanta SC MPL, Beckman Coulter, Fullerton, CA, USA) equipped with a 488-nm argon-ion laser for excitation. Samples were measured at a flow rate of 150–250 cells per second. For each analysis, at least 10,000 events were acquired. The sperm population was gated on the basis of the electronic volume (EV, parameter depending on the cell size) and side scatter signals (SS, parameter depending on cellular granules). The intensity of green (480–550 nm, for FITC) and/or red fluorescence (590–670 nm, for M540, PI) was detected using the FL1 and FL3 channels, respectively. MitoSOX Red fluorescence was detected in channel FL2 (561–550 nm). The fluorescence data were obtained at a fixed gain setting in logarithmic mode (FL1, FL2, FL3). For acquisition and data analysis, the Cell LabQuanta SC MPL Analysis software (Beckman Coulter) was used. Fluorescence reading was repeated two times with distinct samples. Binding specificity was checked with a fluorescence microscope (Olympus) (Fig. 2).

### Statistical analysis

All statistical calculations were performed using the Python 3 with Pandas (https://pandas.pydata.org/ ver 0.24.2), Matplotlib (https://matplotlib.org/ ver 3.0.3), SciPy (https://www.scipy.org/ ver 1.2.1), Seaborn (https://seaborn.pydata.org/ ver 0.11.0) and scikit-posthoc (https://pypi.org/project/scikit-posthocs/ver 0.5.4) libraries as previously described (17, 18). The data distribution was verified using the Shapiro‒Wilk test. As the variables were not normally distributed, the nonparametric Kruskal‒Wallis test followed by the Dunn test with Holm correction was applied to compare the parameters among the studied groups. Correlations were assessed using the Spearman rank test. A *p* < value of 0.05 after the post hoc test was considered significant.

## Results

**Fig. 1:**
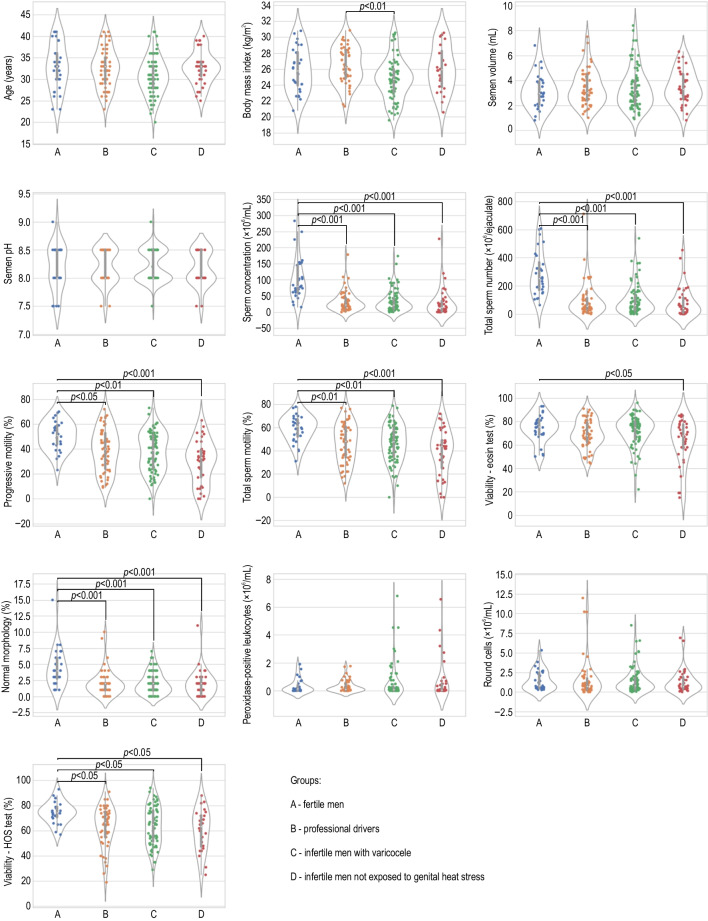
Comparisons of clinical and standard semen parameters. Data are expressed as median, Q1–Q3, and range. Dunn test with Holm’s correction, *p* < 0.05, *p* < 0.01, *p* < 0.001 vs. control or varicocele group; HOS test — hypo-osmotic swelling test

The comparison of age, BMI, and conventional semen parameters among the studied groups is summarized in Fig. 1. In addition, the descriptive statistics (medians, ranges, means ± SDs) of clinical and standard semen parameters are presented in Supplementary Table 1. There was no statistically significant difference in age between fertile men and the studied groups. The BMI of varicocele patients was lower than that of the group of drivers (*p* < 0.01). The results for semen volume, pH, number of peroxidase-positive leukocytes, and number of round cells were similar in the groups under study. Standard sperm parameters such as sperm concentration, total sperm number, and the percentage of normal morphology were significantly reduced in all the studied groups compared to the control (*p* < 0.001). Lower progressive motility and total sperm motility were also found in all the study groups than in fertile men (*p* < 0.001 for infertile men not exposed to heat stress; *p* < 0.01 for infertile men having varicocele in both sperm motility parameters; *p* < 0.05 and *p* < 0.01 for drivers in progressive and total sperm motility, respectively). The percentage of viable sperm in the eosine test was significantly reduced only in the group of infertile men not exposed to prolonged genital heat stress compared to the control group (*p* < 0.05). However, when comparing sperm viability measured in the HOS test, each studied group was different from the control (*p* < 0.05).

**Fig. 2:**
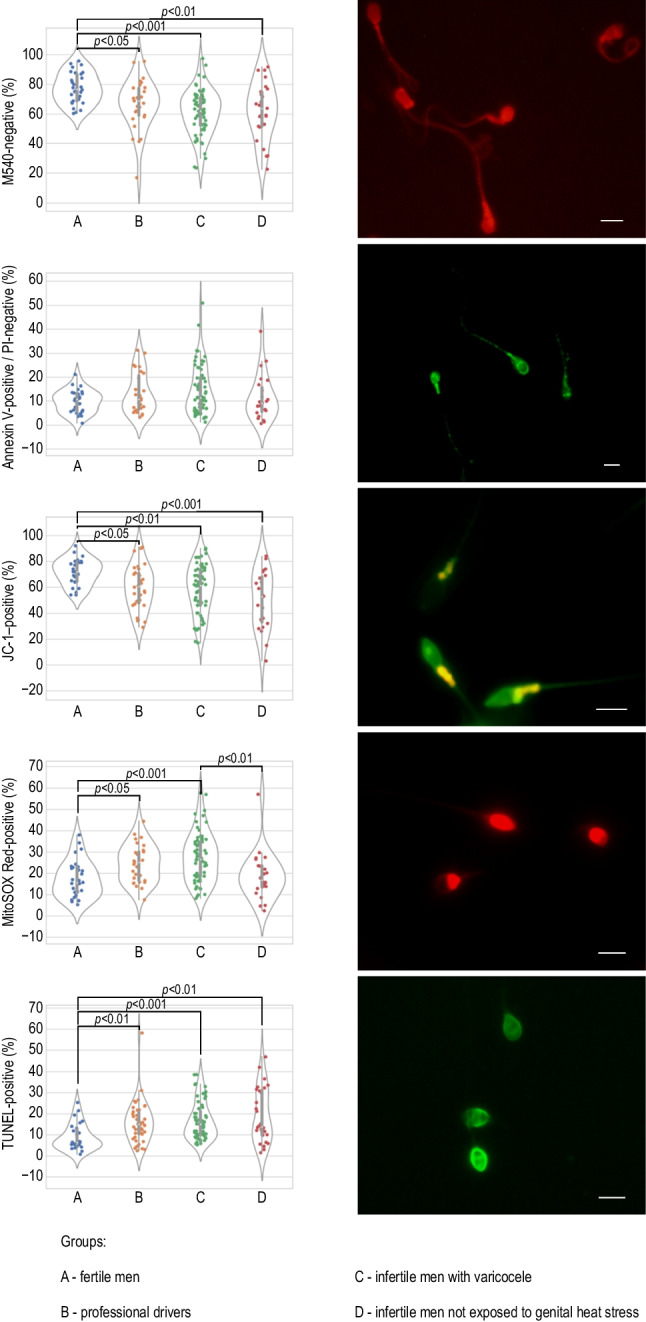
Comparisons of apoptotic and oxidative stress parameters. Data are expressed as median, Q1–Q3, and range. Dunn test with Holm’s correction, *p* < 0.05, *p* < 0.01, *p* < 0.001 vs. control or varicocele group. Representative microphotographs of sperm examined by merocyanine 540 staining, Annexin V-FITC/PI staining, JC-1 staining, MitoSOX Red staining, and TUNEL assay; fluorescence microscope; scale bar = 5 μm

The comparison of sperm apoptotic and oxidative stress parameters among the studied groups is summarized in Fig. 2. In addition, the descriptive statistics (medians, range, means ± SDs) of sperm apoptotic and oxidative stress parameters are presented in Supplementary Table 2. No significant changes in PS externalization in sperm were observed among the groups under study. The percentage of sperm with polarized mitochondria (JC-1–positive sperm) was significantly lower in all the study groups than in the control group (*p* < 0.05, *p* < 0.01, and *p* < 0.001 for drivers, varicocele patients, and infertile men not exposed to heat stress, respectively). Moreover, the decline in mitochondrial membrane potential was accompanied by a significant increase in the level of DNA fragmentation (TUNEL-positive sperm) compared to the value obtained for fertile men (*p* < 0.01 for drivers and infertile men not exposed to heat stress; *p* < 0.001 for varicocele patients). Of the oxidative stress parameters, the percentage of sperm cells without membrane lipid disorders (M540-negative sperm) was significantly lower in all the study groups than in the control group (*p* < 0.05, *p* < 0.01, and *p* < 0.001 for drivers, infertile men not exposed to heat stress, and varicocele patients, respectively). Interestingly, a significant increase in MitoSox Red fluorescence intensity in sperm was observed only in the groups exposed to genital heat stress compared to the control group (*p* < 0.05 for drivers and *p* < 0.001 for varicocele patients). The percentage of MitoSOX Red-positive sperm in the varicocele group was significantly higher than that in the group of infertile men not exposed to heat stress (*p* < 0.01).

**Fig. 3:**
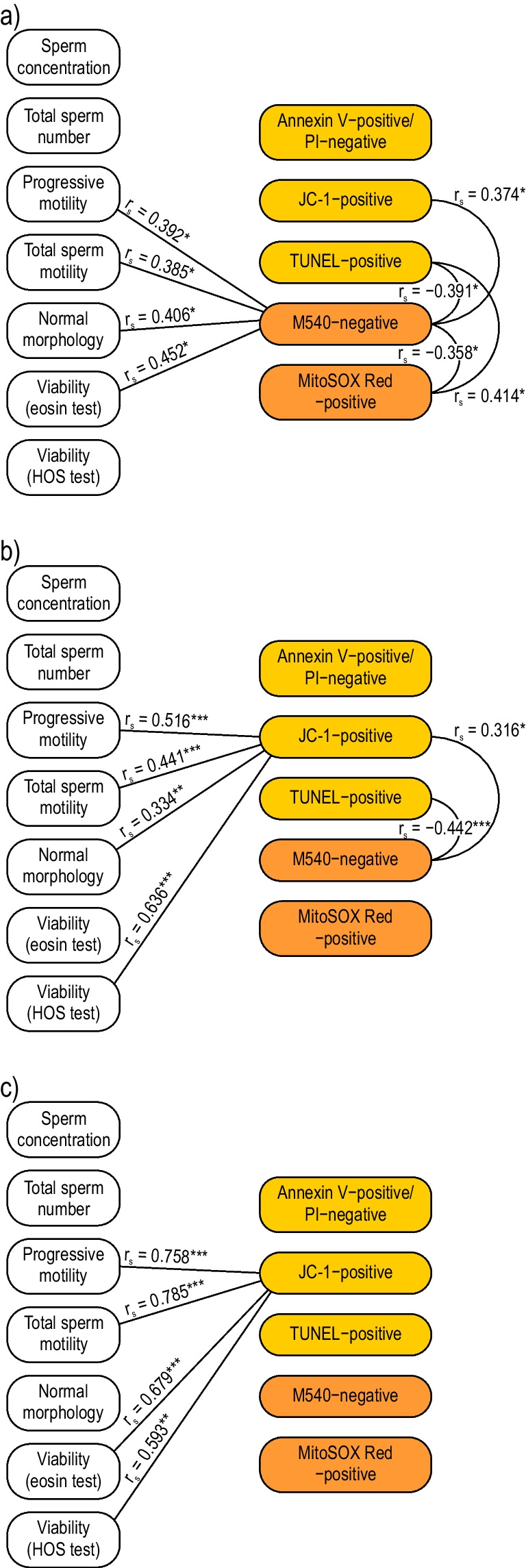
Correlation pleiades of the studied parameters in professional drivers (**a**), infertile men with varicocele (**b**), infertile men not exposed to genital heat stress (**c**). HOS test — hypo-osmotic swelling test; M540 — merocyanine 540; *r*_*s*_ — Spearman’s correlation coefficient, *p < 0.05, **p < 0.01, ***p < 0.001

Figure 3 shows the Spearman rank order correlation values for conventional sperm characteristics and apoptotic parameters in the research groups. There were eight significant correlations in the group of drivers. A decrease in the percentage of sperm without membrane lipid disorders by the M540 test significantly correlated with the deterioration of conventional sperm parameters, such as progressive motility (*r*_*s*_ = 0.392), total sperm motility (*r*_*s*_ = 0.385), viability (*r*_*s*_ = 0.452), and morphology (*r*_*s*_ = 0.406). Additionally, the percentage of M540-negative sperm was positively associated with the percentage of JC-1–positive sperm (*r*_*s*_ = 0.374) and negatively associated with the percentage of MitoSOX Red-positive (*r*_*s*_ =  − 0.358) and TUNEL-positive (*r*_*s*_ =  − 0.391) sperm. Interestingly, a significant positive correlation between the amount of superoxide anion produced in sperm mitochondria and the level of sperm DNA fragmentation (*r*_*s*_ = 0.414) in the group of drivers was also noted. In the group of infertile men with varicocele, the percentage of JC-1–positive sperm was positively correlated with progressive motility (*r*_*s*_ = 0.516), total sperm motility (*r*_*s*_ = 0.441), morphology (*r*_*s*_ = 0.334), and HOS test (*r*_*s*_ = 0.636). Additionally, a decrease in the percentage of sperm with lipid membrane asymmetry visible in the M540 test correlated with an increase in the expression of classic apoptotic markers, such as low mitochondrial membrane potential (*r*_*s*_ = 0.316) and high DNA fragmentation (*r*_*s*_ =  − 0.442). In the group of infertile men not exposed to genital heat stress, the percentage of JC-1–positive sperm was negatively correlated with the deterioration of progressive motility (*r*_*s*_ = 0.758), total sperm motility (*r*_*s*_ = 0.785), viability in the eosin staining (*r*_*s*_ = 0.679), and the HOS test score (*r*_*s*_ = 0.593).

## Discussion

**Fig. 4:**
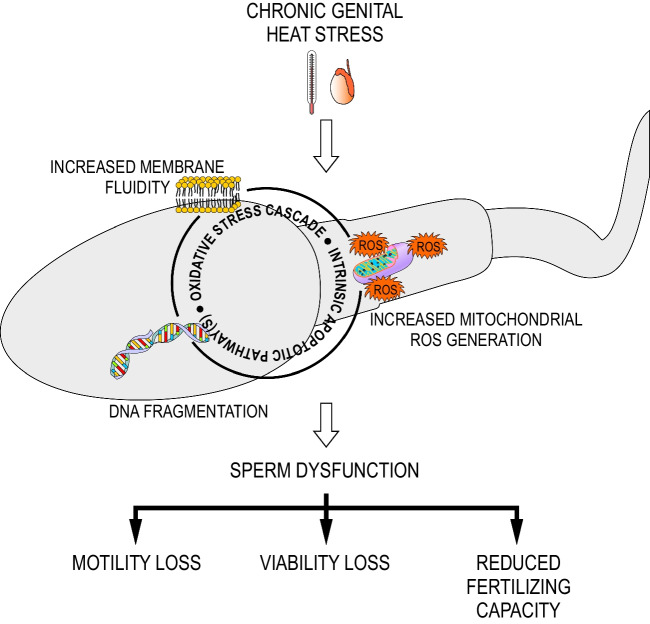
Relationship among genital heat stress, oxidative stress, apoptosis, and subsequent male subfertility/infertility; ROS— reactive oxygen species

This retrospective study constitutes the first attempt to correlate the suggested standard sperm alterations with the sperm oxidative and apoptotic status under real-life conditions of prolonged genital heat stress. The findings support the hypothesis that the intrinsic apoptotic pathway is one of the main mechanisms underlying sperm death in response to the oxidative stress promoted by heat (Fig. 4).

In the present study, we found significant variations in routine sperm parameters in all the studied groups, irrespective of the thermal insult. The data obtained in the group of infertile patients with varicocele confirmed the findings of a meta-analysis by Nork and coworkers [6], which also revealed a significant decrease in sperm density, motility, and/or morphology in men with this pathology compared to normozoospermic and fertile subjects. However, out of the rich available literature on semen quality in varicocele, there are also retrospective cohort studies indicating weakened standard sperm characteristics but with values within so-called WHO reference limits [27]. In the present study, the varicocele group included only infertile patients, 85% of whom had varicocele diagnosed as grade 2 or 3, which skewed our results. Furthermore, we found relatively severe deterioration in standard sperm parameters in the group of professional drivers. These results are in agreement with several previously published clinical reports in which significant alterations in sperm concentration, motility and/or morphological forms in drivers and men with long commuting times were observed [17–19]. Taking into account the significant increase in scrotal temperature while driving [28], it can be concluded that in this occupational group, scrotal heat stress may be a sufficient factor affecting ejaculated sperm quality due to disturbed spermatogenesis.

The present study is a continuation of our previous reports concerning the seminal oxidative stress scavenging system in men exposed to prolonged genital heat stress, which demonstrated low seminal total antioxidant capacity level and high catalase activity in groups of professional drivers and infertile men with varicocele [20, 21]. Taking into account our previous findings, we could postulate that during active scrotal overheating, the dysregulation of seminal antioxidant components is a consequence of heat stress-induced reactive oxygen species generation in sperm. Therefore, adequate levels of low-molecular-weight antioxidants, supporting the seminal antioxidant enzyme defense system, could protect sperm from peroxidative damage under heat. Consistent with our findings and conclusions, some previous experimental studies have shown a reduction in pathological redox levels in human sperm subjected to heat stress in the presence of vitamin C [29] or melatonin [30].

Apoptosis in mature sperm has been a subject of considerable debate in the context of its involvement in the etiology of male infertility, the clinical significance of apoptotic markers, and the postulated link with oxidative stress initiated in the sperm mitochondria [31–33]. In this study, as demonstrated by others, the analysis of classic apoptotic markers demonstrated that heat stress is one of the factors inducing an apoptotic phenotype, characterized by simultaneously decreased mitochondrial membrane potential and enhanced DNA fragmentation in sperm [14, 34]. The levels of sperm PS translocation in the studied groups did not achieve statistical limits compared to those in the control group. However, in human spermatozoa, the role of PS exteriorization is not obvious, and it can also act as a signaling molecule for sperm phagocytosis as well as an element of dynamic changes in the plasma membrane occurring during the process of fertilization [35]. Although we found a sperm apoptotic phenotype more often in all the study groups than the control group, the particular biomarker expression levels seem to depend on the factors that triggered its induction in each population. In men exposed to genital heat stress, a significant increase in mitochondrial superoxide anion generation has been reported. Moreover, only in these study groups did we find a clear set of apoptosis hallmarks, but especially in the group of drivers. Notably, similar relationships of spontaneous mitochondrial superoxide anion generation with membrane fluidity and DNA damage in sperm were previously observed in animal heat stress models, which emphasizes the pathophysiological significance of the present clinical findings [4].

The numerous correlations between sperm membrane fluidity measured by the M540 test, which reflects the degenerative membrane modifications occurring during apoptotic events in human spermatozoa, and standard sperm parameters observed in the group of drivers provided further clear, although indirect, support for the concept that the stimulation of a state of oxidative stress leading to an intrinsic apoptosis pathway may be critical in heat-induced sperm death. However, it cannot be excluded that the observed high numbers of dead/dying sperm, especially in the HOS test, in the study groups also underlined the role of nonapoptotic cell death processes. A previous study documented necrosis associated with peroxynitrate-affected permeability of mitochondrial membranes as a new variant of cell death in human spermatozoa [36]. Although heat stress induces both oxidative and nitrosative reactions, no study reflecting such effects in sperm under scrotal heat stress has been reported. Notably, recent human sperm proteomic studies conducted on men subjected to transient scrotal hyperthermia indicated the role of energy metabolism-related proteins in different death/survival pathways causing oligozoospermia and asthenozoospermia due to scrotal hyperthermia [37]. As for the group of infertile men not exposed to genital heat stress, the present data suggest a rather small role of the intrinsic apoptosis cascade in their severely decreasing sperm quality; however, their data still do not exclude the involvement of sperm mitochondria in the causative pathomechanisms.

We are aware of some limitations of the present study. First, the participants had no scrotal temperature measurements because this procedure was not a part of a routine infertility work-up. However, the original study design and the strict exclusion criteria, especially regarding the contribution of the other known oxidative stress factors, allowed us to reduce selection biases. Second, the research was conducted on a relatively small study population. Larger studies would probably provide even stronger and more reliable statistical relationships between variables. Third, the study was a part of a multicenter research project, and variations in laboratory measurements could limit the validity of some data. It should be highlighted that the collaborative framework included researchers with documented experience in an andrology laboratory and in creating all the protocols used in the study. Standard semen parameters were evaluated by experts in the SpermControl Interlaboratory Semen Test Quality Control Program to ensure proper standardization, and the participants were recruited by urologists with a certificate from the European Academy of Andrology. Last, other biomarkers of sperm apoptosis could have provided an even better picture of genital heat stress sperm death mechanisms.

## Conclusion

This is the first retrospective clinical study to successfully demonstrate that chronic environmental genital heat stress compromises ejaculated sperm quality in humans by intrinsic mitochondria-dependent apoptosis cell death mechanisms. This observation may have some implications for recommendations concerning treatment options in male subfertility/infertility caused or complicated by scrotal hyperthermia, which should aim to protect sperm against chronic oxidative stress followed by DNA damage.

### Supplementary Information

Below is the link to the electronic supplementary material.

Supplementary file1 (PDF 299 KB)

Supplementary file2 (PDF 114 KB)

## Data Availability

Data available on request from the corresponding author.
