# Supplementary material for: The role of the intrinsic pathway of apoptosis in human ejaculated sperm damage under a state of scrotal heat stress
Source: J Assist Reprod Genet. 2023 Dec 8;41(1):99–108. doi: 10.1007/s10815-023-02992-9 (PMC10789687; doi:10.1007/s10815-023-02992-9)
Supplement: Supplementary file 1 — Supplementary file1 (PDF 299 KB) [file 10815_2023_2992_MOESM1_ESM.pdf]

Supplementary Table 1. Descriptive statistics of clinical and standard semen parameters

Standard semen parameter	Fertile men	Professional drivers	Infertile men with varicocele	Infertile men not exposed to genital heat stress
Age (years)	33.00 (23.00–41.00)<br>32.93 ± 5,50<br>n=29	33.00 (23,00–41.00)<br>32.57 ± 4.55<br>n=54	31.00 (20.00–41.00)<br>30.78 ± 4.64<br>n=78	33.00 (25.00–41.00)<br>32.89 ± 4.11<br>n=37
BMI (kg/m^2)	25.79 (20.78–31.92)<br>26.15 ± 3.25<br>n=28	26.30 (21,31–31.00)<br>26.30 ± 2.90<br>n=44	24.73 (19.57–32.42)<br>24.85 ± 2.88<br>n=70	25.92 (20.57–32.32)<br>26.28 ± 3.29<br>n=24
Semen volume (mL)	2.95 (0.80–6.80)<br>3.15 ± 1.39<br>n=29	3.00 (1.00–7.50)<br>3.37 ± 1.44<br>n=54	3.00 (0.90–8.40)<br>3.3 ± 1.66<br>n=78	3.30 (0.80–6.30)<br>3.49 ± 1.36<br>n=37
Semen pH	8.00 (7.50–9.00)<br>8.18 ± 0,39<br>n=27	8.00 (7.50–8.50)<br>8.20 ± 0.30<br>n=44	8.00 (7.50–9.00)<br>8.22 ± 0.28<br>n=69	8.00 (7.50–8.50)<br>8.17 ± 0.30<br>n=32
Sperm concentration ($\times 10^6$/mL)	79.45 (15.50–283.00)<br>102.54 ± 66.32<br>n=29	22.54 (0,22–178.00)<br>33.88 ± 31.86<br>n=54	22.00 (0.20–173.50)<br>33.28 ± 33.66<br>n=78	15.00 (0.03–227.00)<br>29.23 ± 44.34<br>n=37
Total sperm number ($\times 10^6$/ejaculate)	252.90 (64.80–608.00)<br>286.89 ± 157.46<br>n=29	57.47 (1.01–712.00)<br>98.69 ± 118.17<br>n=54	62.80 (0.44–537.80)<br>97.69 ± 103.81<br>n=78	41.40 (0.08–454.00)<br>84.66 ± 109.29<br>n=37
Progressive motility (%)	51.00 (23.00–70.00)<br>50.43 ± 11.90<br>n=29	40.00 (9.00–72.00)<br>39.30 ± 17.24<br>n=54	39.00 (0.00–73.00)<br>37.63 ± 15.80<br>n=78	33.00 (0.00–63.00)<br>29.94 ± 17.32<br>n=37
Total sperm motility (%)	61.00 (32.00–78.00)<br>59.07 ± 11.5<br>n=29	48.50 (12.00–77.00)<br>46.94 ± 16.92<br>n=54	48.00 (0.00–79.00)<br>46.40 ± 16.26<br>n=78	44.00 (0.00–72.00)<br>38.86 ± 19.88<br>n=37
Viability – eosin test (%)	75.50 (50.00–93.00)<br>74.50 ± 11.63<br>n=29	68.00 (44.00–91.00)<br>69.15 ± 12.21<br>n=54	75.00 (22.00–96.00)<br>71.73 ± 13.20<br>n=77	68.00 (15.00–86.00)<br>63.08 ± 19.18<br>n=35
Normal morphology (%)	4.00 (1.00–15.00)<br>4.39 ± 2,98<br>n=29	2.00 (0.00–10.00)<br>2.05 ± 1.98<br>n=54	2.00 (0.00–7.00)<br>2.00 ± 1.69<br>n=78	2.00 (0.00–11.00)<br>2.00 ± 2.12<br>n=35

Peroxidase-positive leukocytes ($\times 10^6$/mL)	0.13 (0.00–1.90)<br>0.33 ± 0,51<br>n=27	0.12 (0.00–1.76)<br>0.32 ± 0.4<br>n=53	0.09 (0.00–6.80)<br>0.54 ± 1.17<br>n=77	0.05 (0.00–6.56)<br>0.64 ± 1.41<br>n=37
Round cells ($\times 10^6$/mL)	0.80 (0.26–5.32)<br>1.53 ± 1,32<br>n=27	0.75 (0.00–12.00)<br>1.59 ± 2.51<br>n=53	0.82 (0.00–8.50)<br>1.46 ± 1.65<br>n=77	0.70 (0.00–6.90)<br>1.31 ± 1.54<br>n=37
Viability – HOS test (%)	74.50 (57.00–93.00)<br>74.58 ± 8,57<br>n=24	66.50 (19.00–91.00)<br>63.31 ± 16.2<br>n=48	65.00 (29.00–94.00)<br>63.83 ± 15.68<br>n=72	63.50 (25.00–88.00)<br>60.62 ± 16.71<br>n=25

Value are median (Min–Max) and mean ± SD
HOS test – hypo-osmotic swelling test; n=number of participants for each variable
