# Supplementary material for: The role of the intrinsic pathway of apoptosis in human ejaculated sperm damage under a state of scrotal heat stress
Source: J Assist Reprod Genet. 2023 Dec 8;41(1):99–108. doi: 10.1007/s10815-023-02992-9 (PMC10789687; doi:10.1007/s10815-023-02992-9)
Supplement: Supplementary file 2 — Supplementary file2 (PDF 114 KB) [file 10815_2023_2992_MOESM2_ESM.pdf]

Supplementary Table 2. Descriptive statistics of apoptotic and oxidative stress parameters

Apoptotic/oxidative stress parameter	Fertile men	Professional drivers	Infertile men with varicocele	Infertile men not exposed to genital heat stress
M540-negative (%)	76.50 (60.09–95.59)<br>77.24 ± 10.46<br>n=29	68.57 (16.71–95.34)<br>66.27 ± 16.62<br>n=29	61.96 (23.31–97.27)<br>60.66 ± 15.40<br>n=64	63.59 (22.41–91.55)<br>61.76 ± 19.06<br>n=25
Annexin V-positive / PI-negative (%)	10.72 (0.69–21.09)<br>9.90 ± 4.78<br>n=27	10.78 (3.16–31.18)<br>13.26 ± 8.43<br>n=27	11.30 (1.16–50.86)<br>14.17 ± 10.36<br>n=60	9.14 (0.54–39.06)<br>11.37 ± 9.33<br>n=23
JC-1–positive (%)	72.00 (54.00–92.00)<br>71.88 ± 10.30<br>n=26	60.50 (29.00–91.00)<br>59.78 ± 16.86<br>n=32	61.50 (17.00–90.00)<br>57.98 ± 18.94<br>n=62	53.00 (3.00–84.00)<br>49.65 ± 22.23<br>n=23
MitoSOX Red-positive (%)	15.69 (5.26–37.91)<br>17.35 ± 8.65<br>n=29	25.18 (7.53–44.30)<br>24.62 ± 8.92<br>n=28	26.45 (8.22–57.59)<br>27.33 ± 11.67<br>n=63	16.81 (2.40–54.95)<br>18.64 ± 11.03<br>n=24
TUNEL-positive (%)	6.40 (0.68–25.27)<br>8.96 ± 6.21<br>n=27	15.03 (2.36–58.13)<br>15.85 ± 9.82<br>n=42	15.01 (5.16–38.52)<br>16.47 ± 8.77<br>n=64	13.76 (1.45–46.96)<br>19.10 ± 12.78<br>n=28

Value are median, Min-Max, mean ± SD

M540 – merocyanine 540; PI – propidium iodide; n – number of participants for each variable
